# The effects of Maraviroc on liver fibrosis in HIV/HCV co-infected patients

**DOI:** 10.7448/IAS.17.4.19643

**Published:** 2014-11-02

**Authors:** Enrique Ortega Gonzalez, Vicente Boix, Miguel Garcia Deltoro, Jose Lopez Aldeguer, Joaquin Portilla, Marta Montero, Emilio Ballester Belda, Vicente Abril, Felix Gutierrez, Carlos Minguez, Josefa Galindo, Concepcion Benirto, Magdalena Garcia Rodriguez, Livia Giner, Purificacion Rubio, Jorge Uso, Giovanna Llerena

**Affiliations:** 1 Infectious Disease Unit, Hospital General Universitario de Valencia, Valencia, Spain; 2 Infectious Disease Unit, Hospital General Universitario de Alicante, Valencia, Spain; 3 Infectious Disease Unit, Hospital La Fe Universitario de Valencia, Valencia, Spain; 4 Infectious Disease Unit, Hospital General Universitario de Alicante, Alicante, Spain; 5 Infectious Disease Unit, Hospital General Universitario de Elche, Elche, Alicante, Spain; 6 Infectious Disease Unit, Hospital General Universitario de Castellon, Castellon de la Plana, Spain; 7 Infectious Disease Unit, Hospital Clinico Universitario de Valencia, Valencia, Spain; 8 Infectious Disease Unit, Hospital General de la Marina Baja, Villajoyosa, Alicante, Spain

## Abstract

**Introduction:**

The fibrogenesis analysis in quimeric CCR1 and CCR5 mice revealed that CCR5 mediates its pro-fibrogenic effects in hepatic cells and promoting stellate cells. The blockage of co-receptors could preserve the progression of hepatic fibrosis in HIV/HCV co-infected patients.

**Objective:**

To evaluate the beneficial effects on hepatic fibrosis in HIV/HCV co-infected patients that are on antiretroviral therapy (ART) with CCR5 co-receptor antagonists.

**Method and materials:**

A multicentre, retrospective pilot study of the evaluation of hepatic fibrosis at mid- and long-term by non-invasive methods in a HIV/HCV co-infected patients cohort in the Valencian Community (Spain) that received ART with a CCR5 co-receptor antagonist. The cut-off points of serum marker tests of hepatic fibrosis were: AST to Platelet Ratio Index (APRI)<0.5 (F0–F1); >1.5 F2; >2 Cirrhosis and Forns Index<4.2 excludes fibrosis; >6.9>F2 fibrosis. Inclusion criteria was established for HIV/HCV co-infected patients on ART with CCR5 co-receptor antagonists that had no previous history of interferon and ribavirin treatment or those who were null-responders and received CCR5 co-receptor antagonist treatment in the previous year. Patients with HBV infection were excluded.

**Results:**

A total of 71 male patients (69%) were reported. A CD4 nadir <100 cells/uL was observed in 42% of patients and 62% (44/71) had a basal CD4 level >350 cells/uL. According to genotypes, 50% were G-1a, 14% G-1b, 11% G-3 and 25% G-4. The median duration of treatment with Maraviroc (MVC) was the following: 45% took it over a year, 41% over two years and 14% over three years. Before starting treatment with MVC, we observed an initial fibrosis of F0–F1 in 49% of patients, F2–F3 in 24% and F4 in 27%. The medium follow-up was of 18.45 months. Progression to a higher fibrosis level was observed in five patients, 11 patients improved at least one stage and the others were stable over time. There were 38 patients taking MVC over two years, 27 patients in this group (59.38%) did not modify their fibrosis, 3 patients (11%) progressed and 8 (29.62%) showed regression of liver fibrosis in one stage.

**Conclusions:**

The data above shows a benefit over fibrosis progression with MVC, expressed by fibrosis serum marker tests in HIV/HCV co-infected patients with CCR5 tropism. The prolong treatment with MVC (over two years) has a better effect on liver fibrosis.

